# Neonatal Infection with Species C Adenoviruses Confirmed in Viable Cord Blood Lymphocytes

**DOI:** 10.1371/journal.pone.0119256

**Published:** 2015-03-12

**Authors:** David A. Ornelles, Linda R. Gooding, C. Garnett-Benson

**Affiliations:** 1 Department of Biology, Georgia State University, Atlanta, Georgia, United States of America; 2 Department of Microbiology and Immunology, Wake Forest School of Medicine, Winston-Salem, North Carolina, United States of America; 3 Emory University School of Medicine, Department of Microbiology and Immunology, Atlanta, Georgia, United States of America; French National Centre for Scientific Research, FRANCE

## Abstract

Credible but conflicting reports address the frequency of prenatal infection by species C adenovirus. This question is important because these viruses persist in lymphoid cells and suppress double-stranded DNA-break repair. Consequently, prenatal adenovirus infections may generate the aberrant clones of lymphocytes that precede development of childhood acute lymphoblastic leukemia (ALL). The present study was designed to overcome technical limitations of prior work by processing cord blood lymphocytes within a day of collection, and by analyzing sufficient numbers of lymphocytes to detect adenovirus-containing cells at the lower limits determined by our previous studies of tonsil lymphocytes. By this approach, adenoviral DNA was identified in 19 of 517 (3.7%) samples, providing definitive evidence for the occurrence of prenatal infection with species C adenoviruses in a significant fraction of neonates predominantly of African American and Hispanic ancestry. Cord blood samples were also tested for the presence of the *ETV6-RUNX1* translocation, the most common genetic abnormality in childhood ALL. Using a nested PCR assay, the *ETV6-RUNX1* transcript was detected in four of 196 adenovirus-negative samples and one of 14 adenovirus-positive cord blood samples. These findings indicate that this method will be suitable for determining concordance between adenovirus infection and the leukemia-associated translocations in newborns.

## Introduction

Leukemia is the most common childhood cancer. Although chromosomal abnormalities associated with childhood acute lymphoblastic leukemia (ALL) often arise before birth [1], the underlying cause of these abnormalities remains unknown [2]. Epidemiological evidence suggests that ALL may be initiated in utero by infection with a common pathogen [3]. Identification of such a pathogen has remained elusive [1,4,5].

Our group published an analysis of Guthrie cards from 49 children who later developed ALL, which identified an increased frequency of adenoviral DNA in leukemic versus normal controls [6]. In that study, the frequency of detection of adenovirus in normal controls, 6%, is in good agreement with the 5.4% detection for adenovirus in amniotic fluid from 1187 sonigraphically normal pregnancies by other investigators [7–10]. However, when we repeated this observation with a larger sample of both leukemic and normal donors, adenoviral DNA was detected in only 2 of a total of 727 samples [11]. Independent testing by other investigators similarly detected adenoviral DNA in Guthrie cards from only 1 of 189 donors [12].

The source of variability of detection of adenoviral DNA in neonatal blood samples is unknown but could arise either from variability in storage conditions of the Guthrie cards or from low numbers of viral DNA-containing cells leading to frequent false negative results. Guthrie cards are a paper substrate to which drops of peripheral blood of newborns are added, dried and stored for decades before sampling for the studies noted above. Guthrie cards may not be handled according to analytical standards required for highly sensitive PCR. As a result, the Guthrie cards could become contaminated by adenoviral DNA and yield a false positive result. To provide more definitive and quantitative evidence for or against a rare, but finite frequency of neonatal infection with human species C adenoviruses, viable human cord blood lymphocytes were collected and analyzed. If storage conditions or low genome copy numbers in past studies limited detection of these viruses, both restrictions should be overcome using relatively large samples of freshly collected material.

In the study described here we use cord blood samples to test for: 1) the presence and amount of adenoviral DNA and, if found, 2) the presence of the most common chromosomal abnormality of childhood ALL, the *ETV6-RUNX1* t(12:21) translocation, in the same samples.

## Materials and Methods

### Clinical samples

Cord blood samples were received from the Grady Memorial Blood Bank under Georgia State University Institutional Review Board exempt approval # H10549. Demographic information for each de-identified sample included the age and apparent ethnicity of the mother and gender of the child.

### Lymphocyte purification

The unused portion of venous umbilical cord blood samples (average 3 mL volume) from 517 infants born at the Grady Memorial Hospital in Atlanta, Georgia were collected in heparin-containing tubes and stored at 4°C. Cells were collected by centrifugation for 15 min at 900 x g, washed with cold RPMI medium, concentrated by centrifugation for 5 min at 400 x g, suspended in 6 ml RPMI and layered on top of 4 ml of Lymphocyte Separation Media (Mediatech, Inc., Herndon, VA). Lymphocytes were harvested from the interface after centrifugation for 20 min at 400 x g (with no brake). After centrifugation, the lymphocyte-containing interface was isolated and washed in RPMI. DNA was isolated from 1/3 of the total number of freshly isolated lymphocytes and the remaining cells were stored in liquid nitrogen for subsequent isolation of RNA.

### DNA Extraction

A median of 1.2×10^6^ (range: 0.5–30x10^6^) lymphocytes were suspended in lysis buffer (0.45% NP-40, 0.45% Tween 20, 2 mM MgCl_2_, 50 mM KCl, 10 mM Tris-HCl [pH 8.3], 0.5 mg per ml proteinase K) at a ratio of 20 μL per 10^6^ cells. The cells were incubated at 55°C overnight with intermittent vortexing. Following the 55°C incubation, proteinase K was inactivated at 95°C for 15 min, insoluble matter was removed by centrifugation, and the DNA-containing supernatant was stored at -20°C.

### Adenoviral DNA detection by nested PCR

Nested PCR for adenovirus was performed using the following primer sets specific for adenovirus species C hexon gene: outer primers: 5’-ATG GCT ACC CCT TCG ATG ATG C–3’, 5’-GCG TTG TAG GCA GTG CC–3’; inner primers: 5’-GAT GCC GCA GTG GTC TTA–3’, 5’-GTC CAG CAC GCC GCG–3’. These primers, previously identified as P11-P14 [13] for nPCR2, generate a 310 bp product. The first round of PCR used 2.5 μL of DNA template in a 25-μL reaction. Following initial amplification using the outer primers for 30 cycles at an annealing temperature of 56°C, 5 μL of the resulting PCR product was amplified in the second round 50 μL PCR reaction using the inner primers for 35 cycles and an annealing temperature of 60°C.

To avoid sample-to-sample contamination, different rooms and dedicated equipment were used for DNA purification and processing, PCR setup, and gel analysis. The PCR setup hood was treated with UV light for 15 min prior to setting up any PCR amplification. Positive-displacement pipettes were used for PCR setup. In the experiments reported here, no signal was detected in any negative control sample. The PCR products were evaluated after electrophoresis through agarose gels by standard methods. DNA was isolated from groups of 5 to 12 cord blood samples. Negative cord blood samples (also run in triplicate) served as an internal negative control as they were the majority in each gel. Five copies of purified Ad2 DNA (Invitrogen) were spiked into the 4^th^ replicate of every sample to confirm that there was no PCR inhibition in the samples. Positive samples identified from the first nPCR (run in triplicate), and a flanking negative sample, were then re-tested by nPCR resulting in 6 nPCR replicates being evaluated for all positive samples.

### Quantitative PCR for adenovirus

Quantitative analysis of species C adenovirus hexon DNA in cord blood lymphocytes was performed using real-time PCR as previously described [13,14]. PCR amplification was carried out in 25 μL reaction mixtures consisting of FastStart Universal Probe Master with Rox (Roche) 0.5 uM of each primer, and 0.3 uM of TaqMan probe. A conserved region of the species C adenovirus hexon gene was detected using the forward primer: 5’- ATG GCT ACC CCT TCG ATG ATG C—3’, reverse primer: 5’- GCG TTG TAG GCA GTG CC—3’ and TaqMan probe: 5’-FAM-CCA CCG AGA CGT ACT TCA GCC T-BHQ-3’. Primers and probes were purchased from Integrated DNA Technologies (Ames, IA). Serial 10-fold dilutions (from 5 x 10^7^ to 5 copies) of purified HAdV-2 DNA (Invitrogen) were used to generate a standard curve for quantitative assessment of cord blood adenoviral DNA content. 2.5 microliters of each DNA sample was used directly in the qPCR assay run on a 7500 Real-Time PCR System (Applied Biosystems). Samples were also tested for glyceraldehyde-3-phosphate dehydrogenase (GAPDH) DNA by real-time PCR using primers as described in [14]. Thermocycling profiles for real-time PCR consisted of 1 cycle of 95°C for 10 min and 50 cycles of 95°C for 15 s, 53°C for 30 s, and 72°C for 35 s in a Bio-Rad iCycler (Bio-Rad). All standards were run in duplicate and test samples were run in triplicate. Negative controls for each quantitative real-time PCR experimental plate included water, the absence of template (in duplicate wells), as well as at least one adenovirus-negative cord blood sample (in triplicate wells).

### RNA Extraction

Frozen cord blood lymphocytes were thawed and washed twice in sterile PBS. The PBS wash was removed and the pelleted cells were homogenized using a QIAShredder column (Qiagen). RNA was then purified using the Qiagen RNeasy Mini Kit. RNA concentration was determined using a NanoDrop spectrophotometer and 200 ng of RNA were used as template for a reverse transcription reaction using DyNamo cDNA Synthesis Kit using random hexamers (Thermo Scientific Catalog #F-470L). Reverse transcription was performed in Bio-Rad Mycycler for 25°C for 10 min; 37°C for 45 min and 85°C for 5 min.

### Detection of *ETV6-RUNX1* translocation

Primers for *ETV6-RUNX1* translocation were modified from standards for the detection of minimal residual disease [15]. The primers used here differ by being longer with a higher T_m_ that is more closely matched for each pair. The pair of nested primers amplify mRNA derived from the prevalent t(12;21) translocation involving a breakpoint within intron 5 of the *ETV6* gene and within the large intron of *RUNX1*. The outer primers were TEL-H: 5’-TGG AGA ATA ATC ACT GCC CAG CGT-3’ (nucleotides 1132–1155 of NCBI Reference Sequence NM_001987) and AML1-G: 5’-GCA TCG TGG ACG TCT CTA GAA GGA TT-3’ (nucleotides 1132–1155 of NCBI Reference Sequence NM_001754). The first product is typically a 334 bp sequence although the exclusion of RUNX1 exon 2 infrequently yields a variant with 39 bp shorter [16]. The inner primers were TEL-I: 5’-TCC GTG GAT TTC AAA CAG TCC AGG CT-5’ (nucleotides 1149–1124 of NM_001987) and AML1-J: 5’-TCG TGG ACG TCT CTA GAA GGA TTC-3’ (nucleotides 288–265 of NM_001754), which amplify a 200 (or 161) bp sequence.

GoTaq Flexi DNA Polymerase (Promega #M8295) was used for both PCRs using a Bio-Rad Mycycler. 5 μL of the 20 μL cDNA product was used as template in the first round 50 μL PCR. Following the first round of amplification using the outer primers for 40 cycles at an annealing temperature of 60°C, 3 μL of PCR product was amplified in the second round 50 μL PCR reaction using the inner primers for 35 cycles and an annealing temperature of 60°C. The UoCB4 cell line was used as a positive control for the *ETV6-RUNX1* fusion gene [17]. RNA samples that were analyzed in Winston-Salem were reverse transcribed with Superscript III (Invitrogen) using random hexamers and conditions recommended by the manufacturer. The integrity of the RNA shipped to Winston-Salem was confirmed with an endpoint PCR with primers 5’-TGT CTG GGT TTC ATC CAT CCG ACA-3’ and 5’-GGC ATC TTC AAA CCT CCA TGA TGC T-3’ spanning exons 3 and 4 of the β-2-microglobulin gene that generates a 248 bp product from the cDNA. Amplification of the TEL-RUNX product was performed as described above.

### Sequence analysis of the *ETV6-RUNX1* amplicon

RT-PCR reaction products were run on a gel and purified with QIAquick Gel Extraction Kit (Qiagen) and submitted to Genewiz (New Jersey) for sequencing with same primers used to generate PCR product.

### Statistical analyses

Statistical analyses were performed with the open source language and environment R [18]. Continuous variables that were normally distributed or normally distributed after log-transformation were compared with the Student t test. Categorical variables were compared with the Fisher exact test. Logistic regression analysis was used to evaluate the association between the presence of adenovirus in the cord blood and age of the mother at birth. A 95% confidence interval (CI) for the fraction of dichotomous variables was calculated according to the method of Agresti and Coull [19] as implemented in the Hmisc package in R [20]. A 2-sided P value of less than 0.05 was considered to indicate statistical significance.

## Results

**Fig 1:**
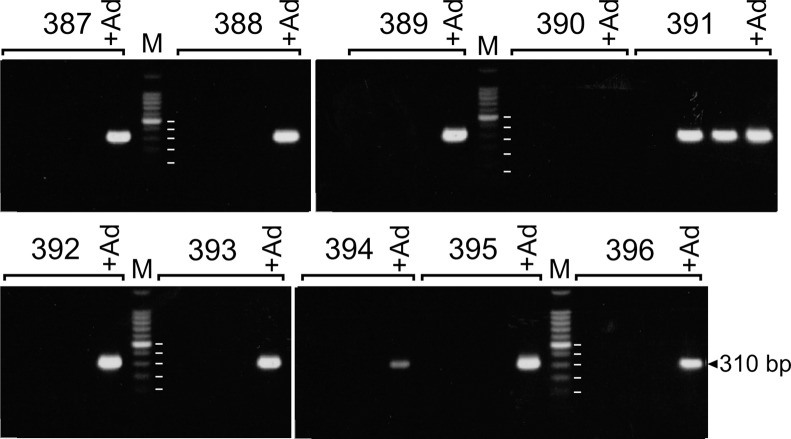
Detection of adenoviral DNA in umbilical vein cord blood. Lymphocytes enriched by Ficoll density gradient centrifugation were evaluated for adenoviral DNA by a nested PCR assay targeting a conserved region of the hexon gene. Four replicates of each numbered cord blood sample were analyzed. As a positive control, five copies of the type 2 adenovirus genome were added to the fourth replicate (+Ad). Lanes indicated by ‘M’ contained a 100-bp ladder reference. The position of DNA standards corresponding to 500, 400, 300, 200, and 100 bp were determined from a brighter exposure and are indicated. Cord blood samples 390 and 391 represent a negative and positive sample, respectively. These samples were reevaluated because of the failure of the positive control (390) and because the diagnostic 310 bp PCR product was observed in only two of three test samples (391). Samples were considered evaluable if the positive control amplified. Samples were considered to contain adenoviral DNA if the appropriate PCR product was observed in at least five of six technical replicates. All positive samples were evaluated on at least two occasions.

**Fig 2:**
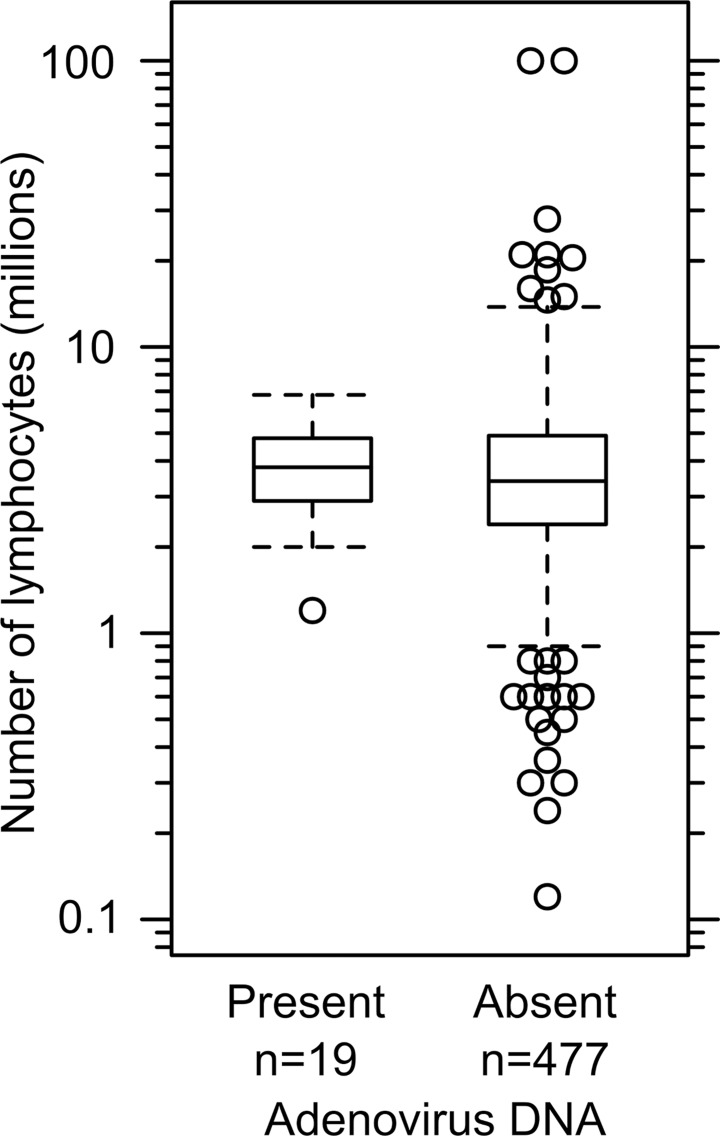
The detection of adenoviral DNA is unrelated to the number of lymphocytes recovered in the cord blood. The number of lymphocytes in cord blood samples that either contain (Present) or do not contain (Absent) adenoviral DNA show similar distributions. Median values of 3.8×10^6^ and 3.4×10^6^ are indicated by the solid horizontal line. The box spans the interquartile range of log-transformed values. Values beyond 1.5-times the interquartile range are plotted as open circles and whiskers indicate the range of values less than 1.5-times the interquartile range.

From September 2010 to July 2011, umbilical vein cord blood of 517 live births at the Grady Memorial Hospital in Atlanta, Georgia was screened for the presence of adenoviral DNA. A nested PCR assay able to detect 5 copies of the species C adenovirus genome identified virus in 19 of 517 or 3.7% of the samples (95% CI: 2.4–5.7%). Samples were considered adenovirus-positive if a PCR product of the diagnostic size was observed in at least five of six technical replicates evaluated in two separate nested PCR experiments (triplicates tested each time). Representative results showing a positive and several negative samples are seen in Fig. 1. The detection of adenoviral DNA was not related to the number of lymphocytes recovered in the sample (Fig. 2) because the median number of lymphocytes recovered from adenovirus-positive cord blood samples was not significantly different from the number of cells in adenovirus-negative samples (p = 0.7). The presence of adenoviral DNA was not associated with the gender of the child (p = 0.5) or with the apparent race of the mother (p > 0.3). Intriguingly, adenoviral DNA was detected more frequently in children born of younger mothers (7% of mothers 20 years or younger versus 2% of mothers over 30 years at birth). Although not significant (p = 0.07), the results suggest a trend towards adenoviral DNA being more frequently found in children born of younger mothers (Table 1). Adenovirus was detected in children of African American and Hispanic ancestry at comparable frequencies (Table 1). To our knowledge, this is the first report of the prevalence of adenovirus in the lymphocytes of newborn children of African American ancestry. The measured frequency of 3.7% is statistically indistinguishable (p = 0.14) from the 5.4% detection for adenovirus in amniotic fluid from 1187 sonigraphically normal pregnancies obtained in the second trimester [7–10]. These results support the notion that adenovirus is fairly common prenatal infection as indicated by the presence of viral DNA in amniotic fluid.

**Table 1:** Adenoviral DNA status as a function of mother’s demographics and infant gender.

	Adenoviral DNA status			
Mother’s ethnicity	Positive	Negative	% positive	95% CI^1^
African/Black	15	396	3.6%	2.2–5.9%
Hispanic	4	77	4.9%	1.9–12%
Caucasian	0	13	0%	0–23%
Asian	0	7	0%	0–35%
Native American	0	1	0%	-
Unknown	0	4	0%	-
Mother’s age				
<16	2	7	22.2%	6.3–55%
16–20	5	92	5.2%	2.2–11.5%
21–25	5	165	2.9%	1.3–6.7%
26–30	5	136	3.5%	1.5–8.0%
31–35	2	73	2.7%	0.7–9.2%
>35	0	25	0.0%	0–13.3%
Gender				
Female	8	253	3.1%	1.6–5.9%
Male	11	245	4.3%	2.4–7.5%
Total				
	19	498	3.7%	2.4–5.7%

^1^The 95% confidence interval for the fraction of adenoviral DNA-positive samples was calculated according to the method of Agresti and Coull [19].

**Fig 3:**
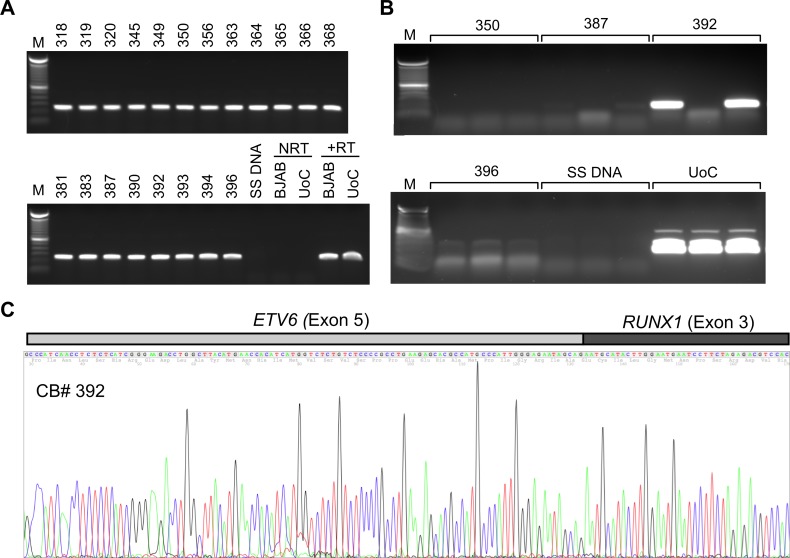
Detection of the *ETV6-RUNX1* fusion transcript by reverse transcription followed by PCR. 200 ng of RNA purified from umbilical vein cord blood lymphocytes was reverse transcribed using random hexamers as primers. (A) The integrity of the RNA in the numbered cord blood samples was confirmed by amplification of a 384 bp sequence spanning the junction of exons 3 and 4 of the β-2-microglobulin gene. Positive controls included RNA from the BJAB and UoCB4 cell lines (+RT). Negative controls included salmon sperm DNA (SS DNA) and the exclusion of reverse transcriptase (NRT). Lanes containing the 100 bp DNA ladder reference are indicated by M. (B) A 200 bp amplicon obtained from a nested PCR assay indicates the presence of the *ETV6-RUNX1* transcript. Representative results show the products of three technical replicates from four numbered cord blood samples, salmon sperm DNA as a negative control, and a 10^-2^ dilution of the *ETV6-RUNX1*-positive UoCB4 cells among BJAB cells as a positive control. Samples in which at least two of three technical replicates were detected from cDNA generated independently in two laboratories and in which no product was observed when reverse transcriptase was omitted were considered to contain the *ETV6-RUNX1* fusion transcript. (C) The *ETV6-RUNX1* amplicon generated from cord blood sample 392 was subjected to sequencing with the primers used to generate the product. The sequencing electropherogram from one of two reactions demonstrates the structure of the expected fusion transcript.

Because adenovirus was definitively detected in some cord blood samples, fourteen adenovirus-positive cord blood samples and 196 adenovirus-negative samples were tested for presence of the most common translocation associated with immature B cell ALL. For this, RNA was extracted from the purified cord blood lymphocytes and reverse transcribed into cDNA. The cDNA was queried with a nested PCR assay designed to detect the *ETV6-RUNX1* (*TEL-AML1*) translocation [15]. Negative controls included RNA from the Burkitt’s lymphoma-derived BJAB cell line, salmon sperm DNA as the template for the nested PCR reaction, and the omission of reverse transcriptase from the cDNA reaction. To serve as a positive control for the *ETV6-RUNX1* translocation, RNA from UoC-B4 cells [17] and BJAB cells was mixed at a ratio of 1:100 and used for reverse transcription. RNA samples were shipped to Winston-Salem for an independent assessment. The integrity of all RNA samples shipped to Winston-Salem was confirmed with an end-point PCR assay to detect the β-2 microglobulin transcript as shown by the representative results in Fig. 3A. cDNA derived from the equivalent of 50 ng of RNA was analyzed in triplicate with the nested PCR assay for the *ETV6-RUNX1* fusion transcript. A sample was considered to contain the target if a PCR product was detected in at least two of three technical PCR replicates performed independently in both Atlanta and Winston-Salem. Representative results showing detection of the *ETV6-RUNX1* fusion transcript in a cord blood sample is seen in Fig. 3B. The identity of each candidate PCR product was determined by sequencing. The sequencing electropherogram (Fig. 3B) confirms the expected fusion between exon 5 of *ETV6* and exon 3 of *RUNX1*. By these criteria, 4 of the 196 adenovirus-negative samples (2.0%, 95%CI: 0.8–5.1%) and one of the 14 of adenovirus-positive cord blood samples (7%, 95% CI: 0.4–32%) contained the *ETV6-RUNX1* translocation (Table 2). Although a higher fraction of adenovirus-positive cord blood samples contained the *ETV6-RUNX1* translocation, a statistically significant difference (p = 0.3) could not be discerned because of the limited number of samples. Although the non-random selection of samples tested for the *ETV6-RUNX1* selection may skew the fraction of cord blood with the fusion transcript, the nested PCR assay used here suggests that over 1% of the children born at Grady Memorial Hospital contained the *ETV6-RUNX1* translocation at birth. It should be noted that this value is statistically indistinguishable from the values ranging between 1% and 4% reported by Eguchi-Ishimae et al. [21], Mori et al. [22], Zuna et al. [23] and Škorvaga et al. [24].

**Table 2:** Detection of *ETV6-RUNX1* fusion transcript in cord blood RNA.

	*ETV6-RUNX1* fusion			
	Present	Absent	% positive	95% CI^1^
Adenoviral DNA				
Positive	1	13	7.1%	0.4–31.5%
Negative	4	192	2.0%	0.8–5.1%
Mother’s ethnicity				
African/Black	3	145	2.0%	0.7–5.8%
Hispanic	2	53	3.6%	1.0–12.3%
Other	0	7	0.0%	0–35.4%
Gender				
Female	2	114	1.7%	0.5–6.1%
Male	3	91	3.2%	1.1–9%
Total				
	5	205	2.4%	1.0–5.5%

^1^The 95% confidence interval for the fraction of *ETV6-RUNX1* fusion transcript-positive samples was calculated according to the method of Agresti and Coull [19].

Although the limited number of samples analyzed in this study do not provide sufficient statistical power to establish an association between the detection of adenoviral DNA and the *ETV6-RUNX1* translocation, it was possible to measure the amount of viral DNA recovered in some of the samples where enough material remained to do so. Five adenovirus-positive cord blood samples were queried by quantitative PCR. Strikingly, the number of viral genomes in the single sample positive for the *ETV6-RUNX1* translocation was 76-fold over the median viral load in the four samples lacking the *ETV6-RUNX1* translocation that could be analyzed (Table 3).

**Table 3:** Adenovirus genome load in selected adenoviral DNA-positive samples.

Cord blood sample	Viral genomes per 10^6^ cells	ETV6-RUNX1 fusion
601	136	-
608	168	-
614	7,664	present
628	136	-
642	32	-

## Discussion

The present study was undertaken to resolve a long-standing conflict in the literature regarding the presence or absence of prenatal species C adenovirus infections in humans. This question is important because these viruses express molecular machinery [25] capable of inducing the genomic translocations that arise prior to birth and lead to leukemia in young children [26–28]. Evidence of prenatal adenovirus infection in samples that also contain the most common translocation of childhood leukemia, *ETV6-RUNX1*, would be strong support for the involvement of adenovirus in the genesis of this disease. Previous studies looking for adenoviruses in amniocentesis fluid, with some exceptions, find viral DNA in about 5% of samples. By contrast, studies using dried peripheral blood spots from newborn infants (Guthrie cards) generally fail to find adenoviral DNA [11,12].

Based on the results reported here we postulate that the negative results cited above are false negatives due to one of the following three technical limitations. Either the initial sample size was too small to contain a single virus-infected cell, or the samples were treated prior to analysis in ways that cause loss of viral DNA, or the cell types being analyzed were not those that harbor latent or persistent adenovirus. The study presented here was designed to overcome these limitations by using freshly harvested material, by using cord blood which is most likely to contain immature fetal cells, and by using a large initial sample size from which to extract virus using a method known to preserve viral DNA in the samples. This study provides unequivocal evidence for the presence of adenovirus genomes in cord blood at birth in 3.7% of newborns.

Species C adenoviruses establish latent infections in mucosal-associated lymphoid tissues [13]. We and other investigators have identified adenoviral DNA in mononuclear cells from adenoids and tonsils of children as young as two years [13,14,29]. By four years of age virtually every child is carrying one or more adenoviral genotypes in their mucosal tissues [13]. Among eight adenovirus DNA-containing tonsil or adenoid samples that were tested, the frequency of virus-containing mononuclear cells ranged from 3 to 3,400 per 10^7^ cells [13]. In addition, the median number of genomes per infected cell is 280 with 95% confidence limits from 15 to 5,000 [13]. The low and variable frequency of virus-containing cells coupled with the likelihood of encountering multiple copies of the genome per infected cell are key to understanding detection of latent adenovirus in human tissue samples.

We detected adenoviral DNA in 3.7% of 517 cord blood samples (Table 1). This frequency agrees with that reported by several groups that evaluated amniotic fluid for adenovirus DNA. Using a PCR assay, Baschat and associates identified adenoviral DNA in 5.4% of amniotic fluid samples [9]. Three additional studies reported detecting adenoviral DNA in approximately 5% of amniocentesis fluid samples from sonographically normal pregnancies [7–10]. In each of these studies, nucleic acid was purified from 2 to 3 mL of whole amniotic fluid using a modification of the one-step method of Chomczynski and Sacchi [30]. These studies did not report removing the cells by centrifugation before testing for adenovirus DNA. By contrast, two studies that sought adenovirus DNA in cell-free amniotic fluid failed to detect viral DNA [31,32]. At mid-gestation, each mL of amniotic fluid contains as many as 10^6^ intact fetal cells [33] and approximately 3,000 genome-equivalents of cell-free fetal DNA [34]. As noted above, latently infected tonsil lymphocytes were found to contain a median of 280 genomes per infected cell [13]. The nested PCR assay used here has a limit of detection of 5 genomes [14]. Thus, a single latently infected cell among 10^6^ cells in one mL of amniotic fluid should contribute sufficient genomes to be detected by the nested PCR assay. Adenovirus purification takes advantage of the fact that progeny virus remains tightly associated with cells and cellular debris [35]. If the same is true in amniotic fluid, removing the cells by centrifugation would effectively remove adenoviral DNA from the sample.

Dried neonatal blood spots also have been screened for fetal pathogens. In contrast to the study of cord blood lymphocytes described here, most studies evaluating neonatal blood spots have failed to detect a variety of viruses [36] including adenovirus [11,12]. The detection of adenoviral DNA in dried blood spots faces several limitations. First, the recovery of viral DNA from dried blood spots may be less efficient than from fresh cells and that efficiency may decrease with time in storage. Second, this process analyzes a limited number of cells. Four 3.2-mm punches from dried blood spots (used in most studies) corresponds to 6 μl of peripheral blood [37] or 24,000 to 65,000 white blood cells, of which one-fourth are lymphocytes. A reasonable probability (>95%) of identifying a cord blood sample as adenovirus DNA-positive requires only three adenovirus-positive cells with a minimum of 20 viral genomes per cell among the million lymphocytes in a typical sample. Using samples punched from dried archival blood spots, we reported in 2010 the detection of adenovirus DNA in only 0.27% (two of 729) of Guthrie card samples [11]. Indeed, if the effective number of cells recovered from the Guthrie card was 30,000 lymphocytes and the true frequency of adenovirus-positive lymphocytes is approximately three in one million, Poisson considerations dictate that we would have detected adenovirus DNA in less than 0.5% of the samples. Third, the blood deposited on the Guthrie cards was obtained from peripheral blood of infants three to four days after birth. The abundance of adenovirus-positive lymphocytes in the peripheral blood at this time likely differs from that in cord blood at birth. Although adenoviral DNA is found in pediatric adenoid and tonsil lymphocytes [13,14,29,38], it is rarely detected in peripheral blood lymphocytes [39,40]. Interestingly, immature T and B cells are more abundant in pediatric adenoids and tonsils than in other secondary lymphoid tissues [41–43]. Cord blood also contains a greater fraction of immature lymphocytes and lymphocytic progenitors than adult peripheral blood [44,45]. Although cord blood and peripheral blood contain similar frequencies of B cells [45], cord blood contains seven-fold more of the fetal B1 cells (CD5+CD19+) than adult peripheral blood [46]. Finally, immature T cells expressing CD38 are more highly represented in cord blood than in peripheral blood [45,46]. If adenovirus infects or persists in immature lymphocytes, it seems plausible that the lymphocytes of the tonsils and adenoids as well as those of cord blood may serve as a preferred reservoir for the virus.

Compelling evidence indicates that chromosomal aberrations found in pediatric leukemias occur before birth [26–28]. Furthermore, an infectious etiology for ALL has been frequently considered [5,47], including a proposal that an otherwise innocuous viral infection in utero precipitates the development of leukemic mutations [48]. Since the *ETV6-RUNX1* translocation is found in nearly 25% of childhood B-cell precursor ALL [49] we analyzed these cord blood samples for an increased frequency of detection of the *ETV6-RUNX1* translocation in adenovirus-containing cord blood lymphocytes. We found that one of 14 (7.1%) adenovirus-positive samples contained the translocation compared to four of 196 (2.0%) adenovirus-negative samples (Table 2). The small number of samples evaluated here does not provide sufficient statistical power to indicate if adenovirus DNA is associated with the *ETV6-RUNX1* translocation. Nonetheless, it is intriguing to note that this single sample (CB 614) also contained the highest viral DNA load among the samples tested (Table 3). Future studies will determine if there is indeed a relationship between the viral DNA load and the presence of preleukemic translocations. A positive correlation would support the notion that an active prenatal adenovirus infection predisposes a child to leukemia. If this is confirmed, it may be appropriate to routinely screen cord blood with a robust and quantitative measure of adenovirus DNA levels.

Overall, we detected the *ETV6-RUNX1* translocation in 2.4% of cord blood samples. This frequency agrees with several reports seeking the *ETV6-RUNX1* translocation in cord blood. Applying both a 5’ nuclease real-time qPCR and a nested endpoint PCR assay to cDNA derived from approximately 10^6^ cord blood lymphocytes, Mori and associates identified the *ETV6-RUNX1* translocation in six of 567 (≈1%) cord blood samples from England [22]. The cord blood of healthy newborns from Prague was analyzed for the presence of the *ETV6-RUNX1*, *MLL-AF4* and *BCR-ABL* fusion transcripts. While the *MLL-AF4* and *BCR-ABL* fusion transcripts were not detected in these samples, five of 253 samples (2%) contained the *ETV6-RUNX1* fusion transcript [23]. Applying fluorescent in-situ hybridization to a specimen with sufficient numbers of cells, these investigators confirmed the presence of cells bearing the chromosomal *ETV6-RUNX1* fusion. A real-time PCR assay identified the *ETV6-RUNX1* fusion transcript in approximately 4% of 200 cord blood samples of children born in Bratislava, Slovak Republic [24]. Although not all studies have identified the *ETV6-RUNX1* transcript in cord blood [50–52], it was identified at a frequency of 1–4% in those that positively detect this fusion transcript.

To our knowledge, the prenatal frequencies of the *ETV6-RUNX1* translocation and adenovirus in the demographic population analyzed here have not been reported. The discovery that three of 148 children of African ancestry (Table 2) contained the pre-leukemic *ETV6-RUNX1* translocation is of interest because children of African or partial African ancestry have a lower risk of developing childhood ALL [53]. It has been suggested that the development of childhood B-cell ALL proceeds in a two-stage fashion, with the initial mutation occurring in utero followed by a second event that promotes the development of frank leukemia [3,48,54]. If our finding that the *ETV6-RUNX1* pre-leukemic translocation arises at comparable frequency in children of African and European ancestry is confirmed with larger numbers of samples, this outcome may inform the search for factors that contribute to the second event purported to drive the development of leukemia.

Adenovirus is unique among animal viruses because its genome exists in actively infected cells, as well as in cell lines used to model adenovirus persistence, as a linear, double-stranded DNA molecule [55]. Although adenoviral DNA is not found in human lymphomas or leukemias [56], adenovirus profoundly suppresses the ability of the infected cell to respond appropriately to double-stranded DNA-breaks. Notably, the ubiquitous species C adenovirus directs the degradation and inactivation of many cellular proteins that sense and repair double-stranded DNA-breaks, thus disabling both the homologous and non-homologous end-joining DNA-repair pathways [25]. Transient expression of the adenovirus oncoproteins elicits chromosomal abnormalities characteristic of the failure to repair double-stranded DNA-breaks in a timely fashion [57,58]. Thus adenovirus has the potential to elicit chromosomal abnormalities in a “hit-and-run” fashion, which is the induction of an oncogenic cellular change followed by loss of the viral genome [57]. Because it is a common prenatal infectious agent of lymphocytic cells, human adenovirus remains a strong candidate for the virus proposed by Smith as an etiologic agent that contributes the initial step toward the development of childhood ALL.
